# Population-level laterality in foraging finless porpoises

**DOI:** 10.1038/s41598-021-00635-6

**Published:** 2021-10-27

**Authors:** Masao Amano, Yudai Kawano, Taketo Kubo, Tsuyoshi Kuwahara, Hayao Kobayashi

**Affiliations:** 1 Graduate School of Fisheries and Environmental Sciences, Nagasaki University, 1-14 Bunkyo-machi, Nagasaki, 852-8521 Japan; 2 Faculty of Fisheries, Nagasaki University, 1-14 Bunkyo-machi, Nagasaki, 852-8521 Japan; 3 Faculty of Bioindustry, Tokyo University of Agriculture, 196 Yasaka, Abashiri, Hokkaido 099-2493 Japan

**Keywords:** Zoology, Animal behaviour

## Abstract

Laterality has been reported in many vertebrates, and asymmetrical cerebral hemisphere function has been hypothesized to cause a left-bias in social behavior and a right-bias in feeding behavior. In this paper, we provide the first report of behavioral laterality in free-ranging finless porpoises, which seems to support the aforementioned hypothesis. We observed the turning behavior of finless porpoises in Omura Bay, Japan, using land-based and unmanned aerial system observations. We found a strong tendency in finless porpoises to turn counterclockwise with their right side down when pursuing and catching fish at the surface of the water. Our results suggest that this population of finless porpoises shows consistent right-biased laterality. Right-biased laterality has been observed in various foraging cetaceans and is usually explained by the dominance of the right eye-left cerebral hemisphere in prey recognition; however, right-biased laterality in foraging cetaceans may have multiple causes.

## Introduction

Laterality is defined as morphological or behavioral asymmetry or functional inequivalence in paired organs in bilaterally symmetric animals. Behavioral laterality has been reported in various animal groups, including cetaceans^1–4^. There are many reports of the right-sided bias in cetacean feeding behavior. The asymmetric wearing of baleen and scratches on the skin of the jaw in gray (*Eschrichtius robustus*) and humpback whales (*Megaptera novaeangliae*) suggests that they feed on their right side when feeding on the seafloor^5–7^. Observations of balaenopterids, including blue (*Balaenoptera musculus*), fin (*B. physalus*), sei (*B. borealis*), and Bryde’s whales (*B. edeni*), have revealed that they usually lunge for prey with their right side down^8^. At several locations in the United States and Mexico, strand feeding common bottlenose dolphins (*Tursiops truncatus*) have been observed to chase fish onto the beach on the right side of their body^9,10^. Similar right-side down feeding postures have been reported in bottlenose dolphins in Florida Keys (plume-feeding)^11^ and in the Bahamas (crater-feeding)^12^. Killer whales (*Orcinus orca*) lunge-feeding for salmon in Kamchatka, Russia, also show the same right-side bias^13^. In general, these behavioral asymmetries are attributed to using the right eye—and thus, the left hemisphere of the brain—to gain and process information regarding prey^12,13^.

**Figure 1:**
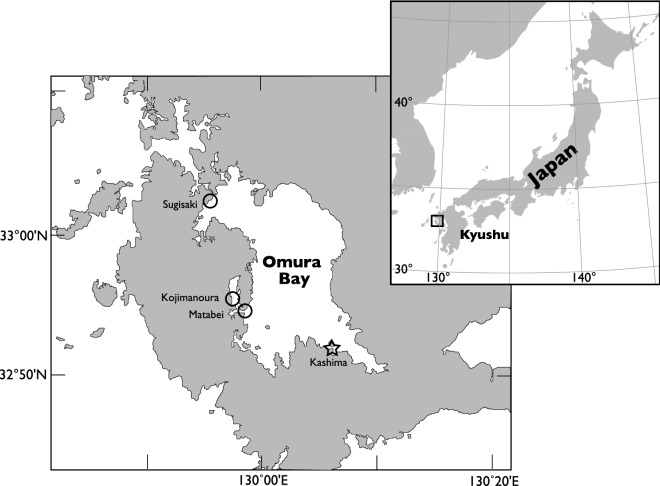
Map of Omura Bay, Japan with the observation sites for monitoring finless porpoise behavior. Star indicates land-based observation site, and open circles indicate aerial observation sites. Map was created using GMT 5.2.1 (https://www.generic-mapping-tools.org).

Here, we report the first case of population-level laterality in narrow-ridged finless porpoises (*Neophocaena asiaeorientalis*), a phocoenid odontocete that inhabits the coastal waters of eastern Asia^14^. This species forms small tentative groups and individuals are usually observed to forage individually at the surface, even if other group members are nearby. A small population of approximately several hundred animals exists in Omura Bay, western Japan (Fig. 1)^15,16^. Porpoises are often observed chasing fish, making sharp turns to catch them at the surface (see Supplementary video). We recorded this behavior using land-based and unmanned aerial system (UAS) observations to examine whether there were any preferences in the turn direction and body posture in these animals. Based on the observations, we discuss the various suggested explanations for the idiosyncratic laterality in cetacean foraging behavior.

## Results

### Land-based observations

We conducted a total of 134 h observation on 22 days. Based on the individual discrimination (see “Methods”), 462 individuals were identified. Among these, 388 porpoises did not show turning behavior, and remaining 74 turned at least once.

**Figure 2:**
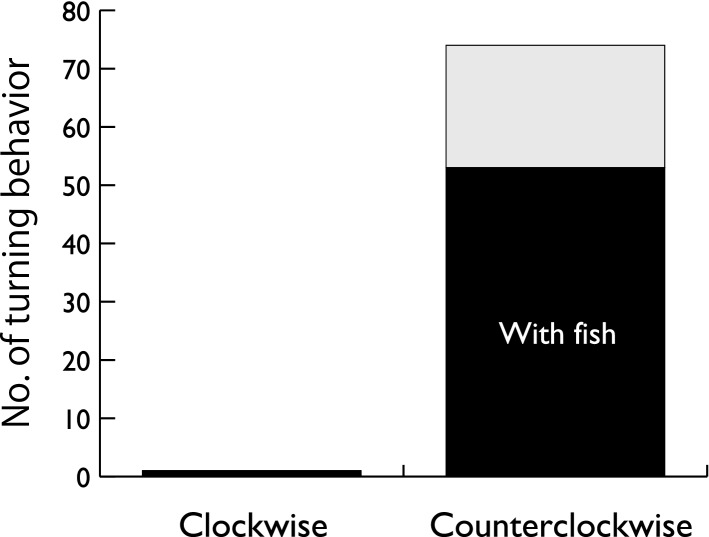
Numbers of clockwise and counterclockwise turning behaviors in finless porpoises noted during land-based observations. Black bars represent cases where a fish or a school of fish was observed ahead of the porpoises.

Of the 74 porpoises, 73 turned counterclockwise (χ^2^(1) = 43.4, P < 0.0001, Fig. 2). We determined body posture in 14 animals, and all of them turned counterclockwise on their right side (χ^2^(3) = 16.8, P = 0.0007). Turning behavior was observed more frequently when a school of or individual fish were observed (53 cases) than when they were not detected (21 cases, χ^2^(1) = 101.5, P < 0.0001, Fig. 2).

### Aerial observations

**Figure 3:**
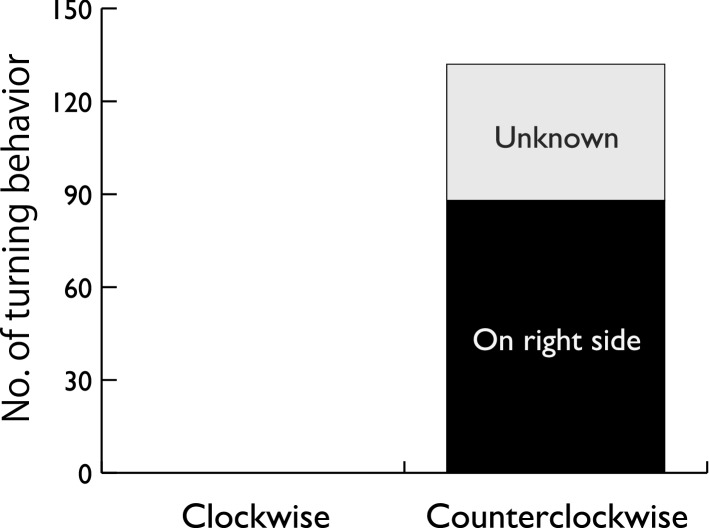
Numbers of clockwise and counterclockwise turning behaviors in finless porpoises noted during aerial observation. Black bars represent the number of observations in which the porpoises turned on their right side. No other postures were observed.

**Figure 4:**
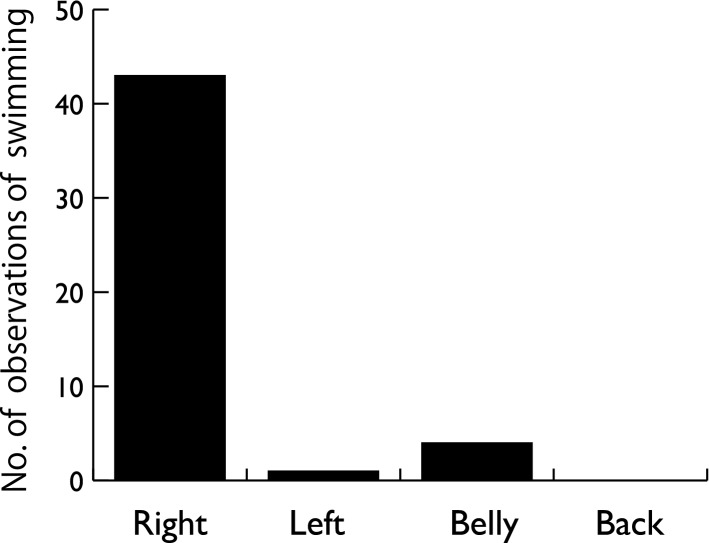
Distribution of postures of finless porpoises in straight swimming before the sharp turn to catch the prey: on the right side (right), on the left side (left), on the belly (belly), and on the back (back).

We conducted 118 UAS flights and obtained 15.5 h of video recordings. The video footage included 132 turning behaviors, and the turning direction was counterclockwise in all cases (Fig. 3, Supplementary video). On 88 occasions, we were able to distinguish the posture of the animal, and all were on their right side. Other postures—that is, belly down, dorsal down, and left side down—were not observed (Fig. 3). Of 48 cases where we were able to observe the posture during straight swimming before the turning behavior, the porpoises were right-side down in 43 cases (χ^2^(3) = 52.4, P < 0.0001, Fig. 4, Supplementary video). We were able to recognize the presence of fish (being pursued by the porpoise) in 25 cases, and fish were observed in front, left, and right of the porpoise in 13, 7, and 1 case, respectively (χ^2^(2) = 6.3, P = 0.043).

## Discussion

In almost all observations, finless porpoises turned in the counterclockwise direction with a right-side-down posture, indicating the existence of population-level laterality in finless porpoises in Omura Bay. Turning behavior was frequently observed in the presence of fish schools in the land-based observations. We also observed instances of individual fish being pursued, where the porpoise made a counterclockwise turn and seemingly caught their prey. These results strongly suggest that the turning behavior of finless porpoises is associated with foraging at or near the surface. This is the first report showing a strong laterality in feeding behavior in Phocoenidae. Further research is required to determine whether this laterality exists in other populations of finless porpoises or other phocoenid species.

Since the dorso-ventral flexibility is larger than lateral flexibility in mammals, a sharp turn with a side-down posture may be a tactic to catch fish, which abruptly change their direction as an escape response. We observed that the finless porpoises were swimming straight on their right side before turning. It is highly likely that porpoises turn counterclockwise in this posture because bending ventrally is much easier and more extensive than bending dorsally in mammals. Thus, a counterclockwise turn during the final attempt to capture prey would be a natural consequence of the right-side down posture when pursuing the prey.

Laterality has been reported in captive Yangtze narrow-ridged finless porpoises^17^, where all six porpoises studied showed a strong tendency to swim around the tank in a clockwise direction. Weaker laterality was observed in the direction of the barrel roll and the side-swimming posture. The results of that study are not directly comparable with those of the current study because the reported laterality was not associated with feeding behavior and the animals were in captivity. However, there are numerous other reports of laterality in foraging behavior among cetaceans, including both mysticetes (e.g., four *Balaenoptera* species^8^, humpback whales^5,7^, and gray whales^6^) and odontocetes (e.g., *Tursiops* spp.^9,10,12^ and Killer whales^13^). In addition to the present case of finless porpoises, all these species adopt right-side down posture when capturing prey, suggesting that this foraging laterality is universal among cetaceans and commonly inherited in this taxon^18^.

A few theories have been suggested to explain this right-side bias in the cetacean foraging behavior: sensory processing (visual processing in particular), laterality in the cerebral hemisphere, asymmetry of echolocation signals, and the morphological laterality in the larynx^12,13^. It is widely accepted that differences in sensory processing between the cerebellar hemispheres cause behavioral laterality in various vertebrates^1^. In vertebrates, the left hemisphere is considered to specialize in controlling routine, internally directed behavior such as feeding. Many vertebrate species also show a rightward bias as they use the right eye to perceive their prey^19^.

Karenina et al.^13^ suggested that cetaceans tend to keep their prey in their right visual field while processing the visual information of the prey in the left cerebellar hemisphere. This explanation was also applied to the laterality of the rolling behavior in blue whales^20,21^. The advantage of the left hemisphere is widely reported in various vertebrates, including fish, amphibians, and birds^22^. However, whether animals tend to use their right eye to see the prey has not yet been established. In the present study, the prey was usually observed in front of the porpoises while pursuing in right-side down posture, though we could not have detected fish on the right side of the porpoise, i.e., deeper in the water. As seen in the UAS footage (Supplementary video), porpoises pursued prey in the ventral, or horizontal, direction (i.e., not to the right or downward direction), as they turned. If the porpoises had tried to see the fish with their right eye during the straight pursuit, they would have had to swim downwards in the final approach, as the right eye was underneath; however, this is not necessarily the case.

When strand feeding, bottlenose dolphins on their right side^9,10^ cannot use their right eye in the final phase of the approach, as the eye is oriented downward and is completely covered when the dolphins beach. Therefore, the current and other observations suggest that cetaceans do not necessarily use the right eye to perceive prey, and the right-side-down posture cannot be entirely explained by the laterality of eye use and visual stimuli processing in the brain.

The asymmetry in echolocation sound beams is another topic of interest. Odontocetes predominantly use the right nasal passage to generate pulse sounds for echolocation^23,24^, and the distribution of the acoustic field is slightly biased toward the right side^24,25^. This is mainly due to the asymmetry in the size of the phonic lips and air sacs associated with sound production^25^. This morphological asymmetry has been considered to be a cause of the behavioral asymmetry of delphinids^12^. However, this asymmetry is much smaller in phocoenids (including finless porpoises) and the asymmetry of the sound beam is biased slightly toward the left, although the difference is not significant^26,27^. Furthermore, the explanation of asymmetry in echolocation signals is only applicable to odontocetes, since mysticetes do not echolocate, at least not in the same way as odontocetes.

In odontocetes, the laryngeal tube penetrates the pharyngeal cavity in a ventrodorsal direction, and food needs to pass through either side of the laryngeal tube. The right piriform sinus is usually larger than the left, and MacLeod et al.^28^ suggested that this is an adaptation to swallow large food items through the relatively wide right passage. However, this may also be a byproduct of asymmetry, such that the right side of the facial region of the skull is larger than the left to accommodate organs for generating echolocation clicks. In either case, when the right cavity is wider, a right-side-down posture may facilitate the passage of food through the right side of the laryngeal tube by gravity. We were unable to determine whether the porpoises swallowed prey immediately after the capture in the same posture; nevertheless, this might be the case because odontocetes with blunt mandibles suck the prey directly into the oral cavity^29^. Our video footage also showed that the porpoises tended to maintain a right-down posture even after capturing prey. However, this asymmetry in the laryngeal tube is not observed in mysticetes which also show right side down posture in foraging behavior.

Asymmetry in the direction of prey escape may be another possibility. Fish preferentially use their right eyes to see predators or baits, which seems to be associated with the visual control of response when a rapid decision should be made^1^. If fish are apt to see a predator with the right eye, they are positioned to the predator's left. Accordingly, the fish receive a stimulus from the predator on their right side, which invokes the reaction (called c- or s-start) to escape in the direction opposite to the stimulus^30^, that is, leftward. If prey fish have this tendency, it is reasonable for cetacean predators to take a right down posture, which makes it easier to make a counterclockwise or ventral turn to catch fish escaping leftward. However, this may not explain the laterality in the bottom-feeding or lunge-feeding mysticetes.

In conclusion, there is no straightforward explanation for the right-side bias in foraging cetaceans, although sensory asymmetry in the neural processes of visual, auditory, or tactile sensory stimuli from the prey is generally accepted. However, the relative position of the prey to the cetacean predator is not identical in different foraging activities as prey are typically below bottom-feeding predators and in front of lunge- and pursuit-feeding predators. There are observations in which dolphins turn in various directions in a single feeding bout, although these have rarely been reported. Therefore, to understand why foraging laterality is prevalent among cetaceans, it is necessary to carefully consider each case of laterality and non-laterality within its context.

## Methods

### Land-based observations

We observed finless porpoises from an observation station on the southeast coast of southeast Omura Bay, Japan (approximately 50 m in altitude) from April to August 2014. The observation area was approximately 0.7 km^2^ and was located between the mainland and Kashima Island (Fig. 1).

When spotting the finless porpoises in the observation area, we recorded the time and the number of individuals and observed their behavior with binoculars. When a porpoise made a horizontal turn, we recorded the direction (clock or counterclockwise), body orientation just before the turn (back or belly up, left or right side down). We defined a turn as a porpoise making a turn of more than 180 degrees in place. We recorded the presence of fish (individuals or a school) ahead of the finless porpoise making a turning behavior to determine whether this behavior was associated with foraging. However, fish under the water surface was not always observable, and not detecting fish does not necessarily mean an absence of them.

To avoid obtaining a series of turning behaviors from a single bout in an individual, we tried to distinguish individuals in an observation session. However, it is difficult to follow the submerged animals and identify individuals in this species that lacks a dorsal fin. Therefore, if an individual dived and another was discovered within 3 min, we assumed it was the same individual, based on the maximum dive duration of the finless porpoise (149 s) recorded using data loggers in China^31^. We recorded only the first turning behavior after an interval of more than 3 min from the previous one. This included the data from the individuals that left and re-entered the observation area after a previous observation.

We examined biases in the direction and body orientation using the chi-square test. Body posture was recorded as one of the following: belly down, dorsal down, left side down, and right side down at the first turn; and the number of observations of each body posture were compared using the chi-square test.

### Aerial observations

We conducted UAS observations from March to December 2019 at three sites on the coast of Omura Bay (Sugisaki, Kojimanoura, and Matabei, Fig. 1). After spotting a finless porpoise from land, we dispatched the UAS (Phantom 4 Pro, DJI, China) and recorded a video of the porpoise from an elevation of 30–135 m above the water surface. We had previously confirmed that the UAS being deployed an elevation of 20 m or higher did not change the behavior of finless porpoises.

We used the recording of only the first turning behavior from each animal. Data from different flights (which were usually separated by 20–30 min) and those obtained after the UAS moved more than 100 m from the previous recording site were assumed to be from different animals and were used in analysis.

As in the land-based observations, when a porpoise made a turn of more than 180 degrees in the video footage, we recorded the direction and body posture of the body just before the turn. In addition, we recorded the body posture during straight swimming just before the turn. When a fish pursued by the porpoise was visible, its relative position to the porpoise (front, left or right) was recorded. Biases in the direction, body posture, and position of the fish were examined using the chi-square test.

We used R v3.5.3^32^ for statistical analyses. This study was approved by the Animal Experiment Committee of Nagasaki University, No. 2006191643.

## Supplementary Information

Supplementary Legends.

Supplementary Video 1.

## Data Availability

The datasets generated during and/or analyzed during the current study are available from the corresponding author on reasonable request.

## References

[1] Vallortigara G, Chiandetti C, Sovrano VA (2010). Brain asymmetry (animal). Wiley Interdiscip. Rev. Cogn. Sci..

[2] Vallortigara G, Rogers LJ (2020). A function for the bicameral mind. Cortex.

[3] Rogers, L. J., Vallortigara, G. & Andrew, R. J. *Divided Brains: The Biology and Behaviour of Brain Asymmetries*. (Cambridge University Press, 2013).

[4] MacNeilage PF (2013). Vertebrate whole-body-action asymmetries and the evolution of right handedness: A comparison between humans and marine mammals. Dev. Psychobiol..

[5] Clapham PJ, Leimkuhler E, Gray BK, Mattila DK (1995). Do humpback whales exhibit lateralized behaviour?. Anim. Behav..

[6] Woodward BL, Winn JP (2006). Apparent lateralized behavior in gray whales feeding off the Central British Columbia coast. Mar. Mamm. Sci..

[7] Canning C (2011). Population-level lateralized feeding behaviour in North Atlantic humpback whales, *Megaptera novaeangliae*. Anim. Behav..

[8] Tershy BR, Wiley DN (1992). Asymmetrical pigmentation in the fin whale: A test of two feeding related hypotheses. Mar. Mamm. Sci..

[9] Hoese HD (1971). Dolphin feeding out of water in a salt marsh. J. Mammal..

[10] Silber GK, Fertl D (1995). Intentional beaching by bottlenose dolphins (*Tursiops truncatus*) in the Colorado River Delta, Mexico. Aquat. Mamm..

[11] Lewis JS, Schroeder WW (2003). Mud plume feeding, a unique foraging behavior of the bottlenose dolphin in the Florida Keys. Gulf Mex. Sci..

[12] Kaplan JD, Goodrich SY, Melillo-Sweeting K, Reiss D (2019). Behavioural laterality in foraging bottlenose dolphins (*Tursiops truncatus*). R. Soc. Open Sci..

[13] Karenina K, Giljov A, Ivkovich T, Malashichev Y (2016). Evidence for the perceptual origin of right-sided feeding biases in cetaceans. Anim. Cogn..

[14] Amano, M. Finless porpoises *Neophocaena phocaenoides*, *N. asiaeorientalis*. in *Encyclopedia of Marine Mammals, *3rd edn (eds. Würsig, B., Thewissen, J. G. M. & Kovacs, K. M.). 372–375. (Academic Press, 2018).

[15] Yoshida H, Shirakihara K, Kishino H, Shirakihara M, Takemura A (1998). Finless porpoise abundance in Omura Bay, Japan: Estimation from aerial sighting surveys. J. Wildl. Manag..

[16] Ogawa, N. Distribution and abundance of finless porpoise, *Neophocaena asiaeorientalis*, off the coasts of Japan. Ph.D. thesis. (Tokyo University of Marine Science and Technology, Tokyo, 2017).

[17] Platto S (2017). Behavioral laterality in Yangtze finless porpoises (*Neophocaena asiaeorientalis asiaeorientalis*). Behav. Process.

[18] MacNeilage PF (2014). Evolution of the strongest vertebrate rightward action asymmetries: Marine mammal sidedness and human handedness. Psychol. Bull..

[19] MacNeilage PF, Rogers LJ, Vallortigara G (2009). Origins of the left & right brain. Sci. Am..

[20] Friedlaender AS (2017). Context-dependent lateralized feeding strategies in blue whales. Curr. Biol..

[21] Torres LG, Barlow DR, Chandler TE, Burnett JD (2020). Insight into the kinematics of blue whale surface foraging through drone observations and prey data. PeerJ.

[22] Ventolini N (2005). Laterality in the wild: Preferential hemifield use during predatory and sexual behaviour in the black-winged stilt. Anim. Behav..

[23] Cranford TW (2011). Observation and analysis of sonar signal generation in the bottlenose dolphin (*Tursiops truncatus*): Evidence for two sonar sources. J. Exp. Mar. Biol. Ecol..

[24] Madsen PT, Lammers M, Wisniewska D, Beedholm K (2013). Nasal sound production in echolocating delphinids (*Tursiops truncatus* and *Pseudorca crassidens*) is dynamic, but unilateral: Clicking on the right side and whistling on the left side. J. Exp. Biol..

[25] Au W, Houser D, Finneran J, Lee W (2010). The acoustic field on the forehead of echolocating Atlantic bottlenose dolphins (*Tursiops truncatus*). J. Acoust. Soc. Am..

[26] Au WWL, Kastelein RA, Rippe T, Schooneman NM (1999). Transmission beam pattern and echolocation signals of a harbor porpoise (*Phocoena phocoena*). J. Acoust. Soc. Am..

[27] Madsen PT, Wisniewska D, Beedholm K (2010). Single source sound production and dynamic beam formation in echolocating harbour porpoises (*Phocoena phocoena*). J. Exp. Biol..

[28] MacLeod CD (2007). Breaking symmetry: The marine environment, prey size, and the evolution of asymmetry in cetacean skulls. Anat. Rec..

[29] Werth AJ (2006). Mandibular and dental variation and the evolution of suction feeding in odontoceti. J. Mamm..

[30] Domenici P, Hale ME (2019). Escape responses of fish: A review of the diversity in motor control, kinematics and behaviour. J. Exp. Biol..

[31] Akamatsu T (2002). Diving behaviour of freshwater finless porpoises (*Neophocaena phocaenoides*) in an oxbow of the Yangtze River, China. ICES J. Mar. Sci..

[32] R Development Core Team. *R: A Language and Environment for Statistical Computing* (2019).

