# Supplementary material for: Population-level laterality in foraging finless porpoises
Source: Sci Rep. 2021 Oct 27;11:21164. doi: 10.1038/s41598-021-00635-6 (PMC8551196; doi:10.1038/s41598-021-00635-6)
Supplement: Supplementary file 1 — Supplementary Legends. [file 41598_2021_635_MOESM1_ESM.docx]

**Supplementary video**

Turning behavior of a finless porpoise feeding on a school of fish (off Matabei on April 28, 2019).
